# Computational Fluid Dynamics (CFD) Modeling and Simulation of Flow Regulatory Mechanism in Artificial Kidney Using Finite Element Method

**DOI:** 10.3390/membranes10070139

**Published:** 2020-07-03

**Authors:** Tuba Yaqoob, Muhammad Ahsan, Arshad Hussain, Iftikhar Ahmad

**Affiliations:** School of Chemical and Materials Engineering, National University of Sciences and Technology, Islamabad 44000, Pakistan; tyaqoob.pse1@scme.nust.edu.pk (T.Y.); arshad.hussain@scme.nust.edu.pk (A.H.); iftikhar.salarzai@scme.nust.edu.pk (I.A.)

**Keywords:** artificial kidney, hemodialysis, membrane, hollow fiber dialyzer, CFD

## Abstract

There is an enormous need in the health welfare sector to manufacture inexpensive dialyzer membranes with minimum dialysis duration. In order to optimize the dialysis cost and time, an in-depth analysis of the effect of dialyzer design and process parameters on toxins (ranging from tiny to large size molecules) clearance rate is required. Mathematical analysis and enhanced computational power of computers can translate the transport phenomena occurring inside the dialyzer while minimizing the development cost. In this paper, the steady-state mass transport in blood and dialysate compartment and across the membrane is investigated with convection-diffusion equations and tortuous pore diffusion model (TPDM), respectively. The two-dimensional, axisymmetric CFD model was simulated by using a solver based on the finite element method (COMSOL Multiphysics 5.4). The effect of design and process parameters is analyzed by solving model equations for varying values of design and process parameters. It is found that by introducing tortuosity in the pore diffusion model, the clearance rate of small size molecules increases, but the clearance rate of large size molecules is reduced. When the fiber aspect ratio (db/L) varies from 900 to 2300, the clearance rate increases 37.71% of its initial value. The results also show that when the pore diameter increases from 10 nm to 20 nm, the clearance rate of urea and glucose also increases by 2.09% and 7.93%, respectively, with tolerated transport of albumin molecules.

## 1. Introduction

During the development of end-stage renal disease (ESRD), a considerable amount of toxins (ranging from small to large size molecules), naturally filtered by human kidneys, begins to accumulate in ESRD patient’s blood. When the patient is suffering from ESRD, hemodialysis is the most inexpensive and effective therapy to remove these solutes (toxins) from the blood. In this therapy, the blood flows from the patient’s body to an extracorporeal circuit that mimics the function of the human kidney with the help of a hollow fiber dialyzer. The hollow fibers are made of semi-permeable porous membranes with an active surface area of 0.8−2.5 m^2^ and a diameter of nearly 200 nm [1]. These fibers allow convective and diffusive transport of uremic solutes, but resist the transport of albumin and blood cells towards the dialysate compartment. Low molecular weight solute (i.e., urea, glucose) transport is governed by diffusion. The transfer of middle molecular weight solutes (i.e., endothelin, β2-Microglobulin, β2-microglobulin, complement factor D, albumin) requires convection (ultrafiltration). This transport phenomenon’s efficiency depends on hollow fiber geometry, membrane characteristics, and operating variables [2,3,4]. 

In the past 30 years, numerous mathematical models have been proposed to mimic the transport phenomena occurring in vivo. Kunitomo et al. performed in-vitro and in-vivo experiments with polymethyl methacrylate (PMMA) hollow fiber units. He established that post-dilution of blood to compensate for the excessive removal of fluid is the most effective way to enhance the clearance of middle size molecules [5]. Jaffrin et al. and Chang et al. developed a one-dimensional model for combine diffusive and convective transport of solutes through membranes. In-vitro verification of the model shows that values of urea clearance are closer to the experimental result [6,7]. Werynski et al. have reviewed the one-dimensional convection-diffusion model, typically used to explain mass transport in membrane equipped clinical devices [8]. He concluded that the one-dimensional model is not applicable for studying the impact of module geometry and shape on clearance efficiency. Wüpper et al. theoretically analyzed the clinical data to determine density changes in radial direction and change in the concentration of large molecules in an axial direction [9]. Annan et al. presented a two-dimensional axisymmetric model to analyze the effect of mismatch flow in the blood and dialysate compartment [10].

The previous studies have presented a simplified description of solute transport across the membrane by assuming uniform convective flux that permits to solve the model equations analytically [5,6,7]. However, the analytical solution provides the results only at the inlet and outlet of the hollow fibers. Therefore, in the current study, a CFD model is solved with the finite element method that provides solutions on a large number of points present in the computational domain. Some mathematical models established the solute transport from blood to the dialysate side and across the membrane with an overall mass transfer coefficient [4,5,7,8,9,10,11,12,13,14,15,16]. The use of the overall mass transfer coefficient without considering the tortuosity and porosity of porous media introduces the difference between the in vitro and in silico clearance rates. To fill this void, TPDM is used in this study that incorporates the effect of membrane tortuosity and porosity to give better estimation of overall mass transfer coefficient [17]. 

In this study, a two-dimensional axisymmetric mathematical model was developed to simulate the convective and diffusive transport of low molecular weight (LMW) solutes, i.e., urea and glucose and middle molecular weight (MMW) solutes, i.e., endothelin and β2-Microglobulin inside the dialyzer. Mass transfer in blood and dialysate compartment was modeled with convection diffusion equations. The blood and dialysate compartments were coupled with a multi-layer membrane by using TPDM. Computational analysis is performed with the finite element method to figure out those factors that play a vital role in enhancing the dialyzer clearance. Numerical results showed that the clearance efficiency of the dialyzer could be improved by increasing the blood and dialysate flow rate, and the fiber aspect ratio; but the clearance of large size molecules (i.e., endothelin, β2-Microglobulin, β2-microglobulin, complement factor D, albumin) does not increase much due to tortuosity τ of the porous medium. The enhanced clearance efficiency will ultimately reduce the dialysis cost and duration. 

## 2. Mathematical Modeling 

### 2.1. Development of a Model

**Figure 1:**
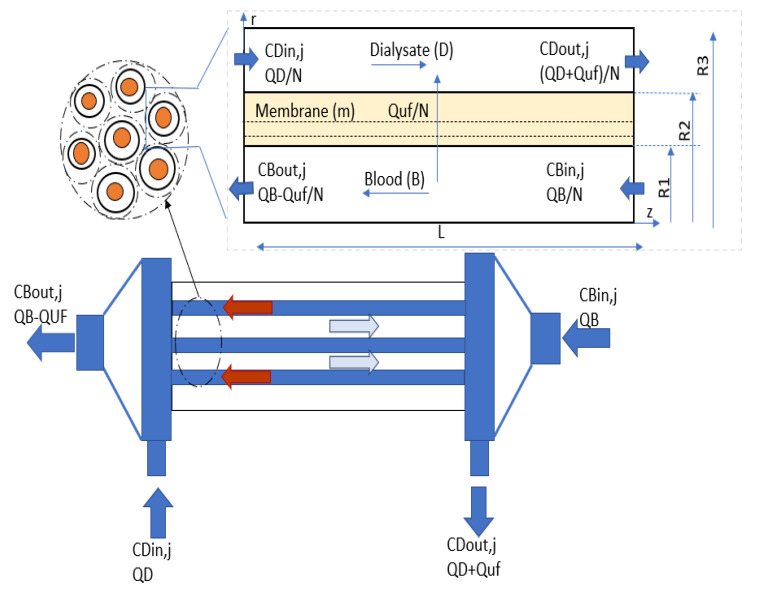
A framework of the geometry of the dialyzer module (lower panel) with its model developed in this work (upper panel).

A framework of the hollow fiber module is presented in Figure 1. A collection of about 12,000 hollow fibers is enclosed in an external shell. Blood passes through the cavity of hollow fibers and dialysate, an aqueous solution of electrolytes, circulates counter-currently at the exterior of the fibers. The transfer of molecules between the blood and dialysate compartment and across the semi-permeable membrane is governed by diffusion and convection. In the presented model, the fibers are assumed to be uniformly spaced, organized in a hexagonal order, and interstice among the adjacent annuli are neglected. It is essential to mention that uneven spacing among fibers would lower the overall mass transfer coefficient on the shell side, leading to a decline of dialyzer efficiency due to the non-uniform distribution of dialysate streams therein. However, the increase of the dialyzer flow rate in some areas of the shell side partially counterbalances the impact of non-uniform distribution in other areas. The solutes considered to study the transport phenomena inside the dialyzer are shown in Table 1.

In this model, a two-dimensional transport of mass and momentum across a three-layer isotropic semi-permeable membrane with a skin, middle and bulk layer is considered. The velocity profiles on both the blood and dialysate side are portrayed with the Navier-Stokes equations [18]. Steady-state, isothermal conditions (T = 37 °C) and laminar flow prevail on both blood and dialysate side with high dilution of solutes [20,21]. It is assumed that the viscosity of both blood and dialysate does not change with applied share. Therefore, these fluids are considered incompressible and Newtonian fluids.

The governing equations and boundary conditions which describe momentum and mass transport in blood and dialysate compartments and across the membrane are as follows:

### 2.2. Governing Equations—Blood Side (i = B)

A cylindrical coordinate system with two dimensions (*r* and *z*) is considered, where dialyzer length (i.e., 0≤z≤L) is taken along the z-direction and radius $\left(i.e.,\;0\leq r\leq r_{3}\right)$ is taken along r-direction. The steady fully developed flow of blood can be described with the continuity equation (Equation (1)) and the Navier Stokes equation (Equations (2) and (3)). Equations (2) and (3) are written for radial and axial velocity components, respectively.

(1) $$\frac{1}{r}\frac{\partial }{\partial r}\left({\mathrm{rv}}_{i}\right)+\frac{\partial u_{i}}{\partial z}=0$$

(r)

(2) $$v_{B}\frac{\partial v_{B}}{\partial r}+u_{B}\frac{\partial v_{B}}{\partial z}=\frac{-1}{\rho_{B}}\frac{\partial P_{B}}{\partial r}+\frac{\mu_{B}}{\rho_{B}}\left[\frac{1}{r}\frac{\partial }{\partial r}\left(r\frac{\partial v_{B}}{\partial r}\right)-\frac{v_{B}}{r^{2}}+\frac{\partial^{2}v_{B}}{\partial z^{2}}\right]$$

(z)

(3) $$v_{B}\frac{\partial u_{B}}{\partial r}+u_{B}\frac{\partial u_{B}}{\partial z}=\frac{-1}{\rho_{B}}\frac{\partial P_{B}}{\partial z}+\frac{\mu_{B}}{\rho_{B}}\left[\frac{1}{r}\frac{\partial }{\partial r}\left(r\frac{\partial u_{B}}{\partial r}\right)+\frac{\partial^{2}u_{B}}{\partial z^{2}}\right]$$

At z = 0 and $0<r<r_{1}$, a fully developed inlet velocity profile for N number of fibers obtained by solving Equations (1)–(3) is:

(4) $$v_{B}\left(r\right)=0{\;\mathrm{and}\;u}_{B}\left(r\right)=\frac{2Q_{B}}{{N\pi r}_{1}^{2}}\left[1-{\left(\frac{r}{r_{1}}\right)}^{2}\right]$$

In Equation (4), $Q_{B}\left(\mathrm{mL}/\min\right)$ is the blood flow rate in each of the hollow fiber and ${\pi r}_{1}^{2}$ is a cross-sectional area of the fiber. Equations (5) and (6) represent that the axial velocity is maximum at r = 0 and no-slip conditions prevail at the walls of the membrane, respectively.

(5) $$v_{B}=\frac{\partial u_{B}}{\partial r}=0\;at\;r=0\;;\;0\leq z\leq L$$

(6) $$v_{B}=u_{B}=0$$

The convection-diffusion equation that governs the mass transfer of solutes *s* present in the blood is:

(7) $$u_{B}\frac{\partial c_{s}}{\partial z}+v_{B}\frac{\partial c_{s}}{\partial r}=D_{s}\left(\frac{\partial^{2}c_{s}}{\partial r^{2}}+\frac{1}{r}\frac{\partial c_{s}}{\partial r}+\frac{\partial^{2}c_{s}}{\partial z^{2}}\right)$$

Here, $c_{s}\left(\mathrm{kg}/m^{3}\right)$ and D_s_(m^2^/s) are the concentration and the bulk diffusivity of solutes *s*, respectively. The boundary conditions to solve Equation (7) are:

$$Ɐz\;\mathrm{at}\;r=0\;\mathrm{and}\;r=r_{1\;}c_{s,i}\left(r,0\right)=c_{s,\mathrm{in}\;}$$

and

$$\frac{\partial c_{s,i}}{\partial r}=0\;\mathrm{where}\;i=B$$

### 2.3. Transfer of Solutes across the Multilayer Membrane (j = Skin, Middle, Bulk)

During the dialysis process, the thin porous membrane selectively allows the low molecular weight solutes to diffuse into a low concentration region. The flux of solutes is proportional to the concentration gradient. The general equation to calculate the solute flux across the membrane is:

(8) $$J_{s}=K_{s}\left(C_{s,B}-C_{s,D\;}\right)$$

Here, J_s_ ($m^{3}/m^{2}s)$ and $K_{s}\left(m/s\right)$ presents the solute *s* flux across the membrane and membrane overall mass transfer coefficient, respectively. $C_{s,B}$ and $C_{s,D}$ are the concentration of solute s in the blood and dialysate compartment. Considering the boundary layers on each side of the membrane the interfacial resistances can be taken in series as:

(9) $$\frac{1}{K_{s}}=\frac{1}{k_{s,B}}+\frac{1}{k_{s,\mathrm{mj}}}+\frac{1}{k_{s,D}}$$

Here, $\frac{1}{k_{s,B}}$ and $\frac{1}{k_{s,D}}$ account for the blood and dialysate side boundary layer resistance, respectively. $\frac{1}{k_{s,\mathrm{mj}}}$ presents the resistance offered by three consecutive layers of membrane. In order to calculate the mass transfer coefficients of blood and dialysate sides, i.e., $k_{s,B\;}\left(m/s\right)$ and $k_{s,D}(m/s$), following a generic correlation was used [21]. For annulus, the hydraulic diameter was used for the calculation of the Reynold number.

(10) NSh,i=1.62(NRe,iNSc,idiz)1/3 where i=B,D

and

(11) NRe,i=uidiρiμi ; Sci=μiρiDi

Here, $N_{\mathrm{Sh},i}$, NRe,i and $N_{\mathrm{Sc},i}$ are presenting Sherwood number, Reynold number and Schmidt number, respectively. 

### 2.4. Tortuous Pore Diffusion Model (TPDM) for Membrane Transfer Coefficient

The mass transfer coefficient of solute s in jth layer of the membrane $k_{s,\mathrm{mj}\;}\left(m/s\right)$ is determined by TPDM. The transfer of solutes *s* within the membrane is hindered by the tortuosity and porosity of the multi-layer membrane. Actually, the pores do not present a straight path for molecules, and its tortuosity quantifies the curved shape of the path. Tortuous pore diffusion model (TPDM) used to account for all the hindrance causing factors of the porous medium is presented below.

(12) $$k_{s,\mathrm{mj}}=\frac{D_{\mathrm{es},j}}{\delta_{j}}$$

(13) $$D_{\mathrm{es},j}=\left(\frac{D_{s,i}{ɛ}_{\mathrm{mj}}}{\tau }\right)F\left(p\right)H_{D}$$

where,

(14) $$F\left(p\right)=\frac{1-2.1050p+2.0865p^{3}-1.7068p^{5}+0..72603p^{6}}{1-0.75857p^{5}}$$

(15) $$p=\frac{R_{s}}{R_{p}}$$

(16) $$H_{D}={\left(1-p\right)}^{2}$$

The Equation (13) presents the tortuous pore diffusion model (TPDM) used to calculate the effective diffusivity $D_{\mathrm{es},j}$ (m^2^/s) of solutes *s* in the porous medium which is less than the bulk diffusivity $D_{s,i}$ (m^2^/s). Friction coefficient F(p) account for the friction that exists between the pore wall and the solute molecules, and p is the ratio of solute radius $R_{s}$ to the pore radius $R_{p}$. The steric hindrance factor $H_{D}$ presents the volume fraction available for the solute molecules in the cylindrical pore. Tortuosity τ defined by the ratio of pore length to the membrane thickness and the experimentally determined values of tortuosity were taken from Yamamoto et al. [17]. ${ɛ}_{\mathrm{mj}}$ presents the porosity of jth layer of the membrane and its experimentally determined values were taken from Islam et al. [22].

### 2.5. Governing Equations—Dialysate Side (i = D)

In hollow fiber dialyzer, the fibers were surrounded by a uniform annulus, as shown in Figure 1. The radius of the annulus r_3_ is larger than the fiber radius r_1._ The velocity of dialysate is also determined by solving continuity Equation (8) and Navier Stokes Equations (9) and (10) with specified boundary conditions of $u_{z}=0$ at *r* = 0 and *r* = $r_{2}$. Here, *r_2_* is the outer radius of the membrane.

(17) $$\frac{1}{r}\frac{\partial }{\partial r}\left({\mathrm{rv}}_{i}\right)+\frac{\partial u_{i}}{\partial z}=0$$

(r)

(18) $$v_{D}\frac{\partial v_{D}}{\partial r}+u_{D}\frac{\partial v_{D}}{\partial z}=\frac{-1}{\rho_{D}}\frac{\partial P_{D}}{\partial r}+\frac{\mu_{D}}{\rho_{D}}\left[\frac{1}{r}\frac{\partial }{\partial r}\left(r\frac{\partial v_{D}}{\partial r}\right)-\frac{v_{D}}{r^{2}}+\frac{\partial^{2}v_{D}}{\partial z^{2}}\right]$$

(z)

(19) $$v_{D}\frac{\partial u_{D}}{\partial r}+u_{D}\frac{\partial u_{D}}{\partial z}=\frac{-1}{\rho_{D}}\frac{\partial P_{D}}{\partial z}+\frac{\mu_{D}}{\rho_{D}}\left[\frac{1}{r}\frac{\partial }{\partial r}\left(r\frac{\partial u_{D}}{\partial r}\right)+\frac{\partial^{2}u_{D}}{\partial z^{2}}\right]$$

The fully developed axial velocity profile of dialysate is:

(20) $$v_{D}=\frac{2Q_{D}}{N\pi \left(\frac{3r_{3}^{4}}{4}+\frac{r_{2}^{4}}{4}-r_{2}^{2}r_{3}^{2}-r_{3}^{4}\ln\left(\frac{r_{3}}{r_{2}}\right)\right)}\left[r^{2}-r_{2}^{2}-2r_{3}^{2}\;\ln\left(\frac{r}{r_{2}}\right)\right]$$

Here, $v_{D}\left(m/s\right)$ and Q_D_ (mL/min) are representing the velocity and volumetric flow rate of dialysate, respectively. The governing equation for dialysate side of solutes *s* transport can be written similar to the Equation (7):

(21) $$u_{D}\frac{\partial c_{s}}{\partial z}+v_{D}\frac{\partial c_{s}}{\partial r}=D_{s}\left(\frac{\partial^{2}c_{s}}{\partial r^{2}}+\frac{1}{r}\frac{\partial c_{s}}{\partial r}+\frac{\partial^{2}c_{s}}{\partial z^{2}}\right)$$

After simulating the mathematical model, the efficiency of the dialyzer (artificial kidney) was determined by calculating the clearance rate of toxins. The dialyzer clearance rate is measured by the following Equation [22]:

(22) $${\mathrm{Cl}}_{s}=\frac{Q_{B}\left(c_{s,\mathrm{in}}-c_{s,\mathrm{out}}\right)}{c_{s,\mathrm{in}}}$$

### 2.6. Computational Method

**Figure 2:**
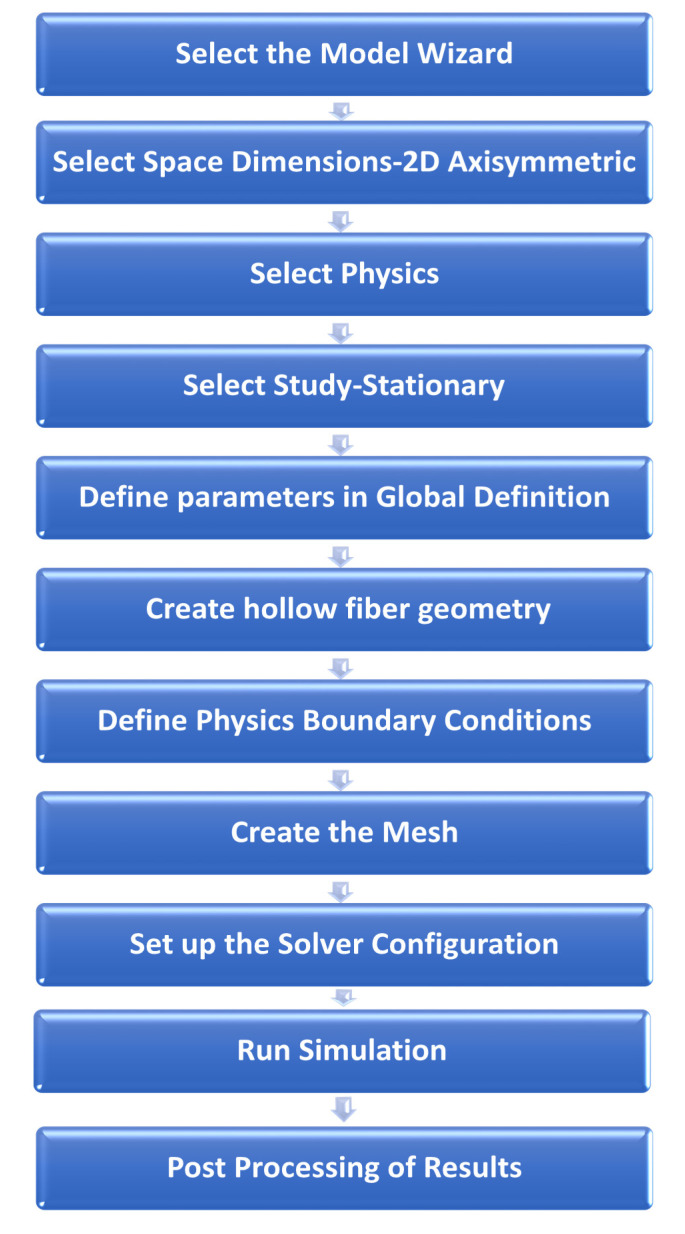
Simulation workflow in COMSOL Multiphysics 5.4.

For numerical integration of the mathematical model, the finite element method was applied with COMSOL Multiphysics 5.4. Free triangular meshing was used to perform the discretization of the computational domain with more than 40,000 triangular elements. The maximum and minimum element size was kept 2.1 × 10^−4^ and 9 × 10^−7^, respectively, with a maximum growth rate of 1.3 and a curvature factor of 0.3. At the blood and dialysate inlets and outlets, and at the interfaces of the membrane with blood and dialysate compartment, local mesh refinement was applied due to the higher complexity of model equations in these areas. Two study nodes were included in the solver configuration, i.e., fully coupled and direct. Fully coupled node combines multi-physics domains, i.e., blood, dialysate, and different membrane layers, while applying the Newton’s method damped version. Under direct node MUMPS (multifrontal massively parallel sparse) method was chosen to enhance the computational efficiency. This method performs the factorization of linear systems in the form of Ax = b, where matrix A is factorized to determine the solution ‘x’. By using the literature-reported values of model parameters, as listed in Table 2, steady-state 2D profiles of velocity and solute concentration were determined. In order to determine the solutes *s* concentration at the outlet of the fiber, the surface average was taken at the outer cross-sectional area. The solution procedure followed to solve the CFD model using Finite Element Method solver is shown in Figure 2.

### 2.7. Validation

**Figure 3:**
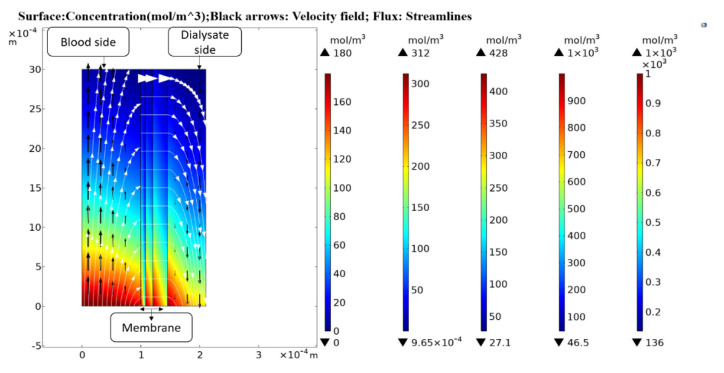
Axisymmetric concentration contour of urea at both blood and dialysate side and across the membrane.

The mathematical model developed was simulated in COMSOL Multiphysics with inlet, outlet, and boundary conditions. The concentration contour of urea in the blood and dialysate compartment and across the membrane is shown in Figure 3.

**Figure 4:**
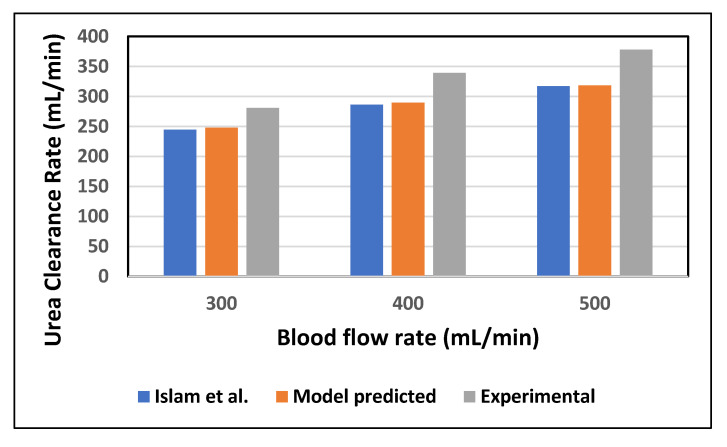
The urea clearance rate for in-silico and in-vitro cases at varying blood flow rate with constant dialysate flow rate (Q_D_ = 500 mL/min).

In order to validate the proposed mathematical model, the model-predicted urea clearance rate was compared with Islam et al. (in-silico) [22] and experimental data reported in the literature at increasing blood flow rates [23]. In Figure 4, the clearance rate of urea is compared with Polyflux 210H data provided by the manufacturer [23]. The values of the experimental clearance rate, in Figure 4, are reported in the literature with the combined effect of diffusion and ultrafiltration. In Table 3, the percentage difference between experimental data and the model predicted values is because the ultrafiltration flux across the membrane was not included in the current model. However, the model predicted values for diffusive transport of solute across the membrane are in good agreement with the Islam et al. (in silico) results. 

## 3. Results and Discussion

The aim of developing this mathematical model was to investigate the impact of module geometry and operating conditions on clearance efficiency and to provide a model that can be simulated at different values of parameters to optimize the clearance rate.

### 3.1. Effect of Operating Conditions on Clearance Efficiency

**Figure 5:**
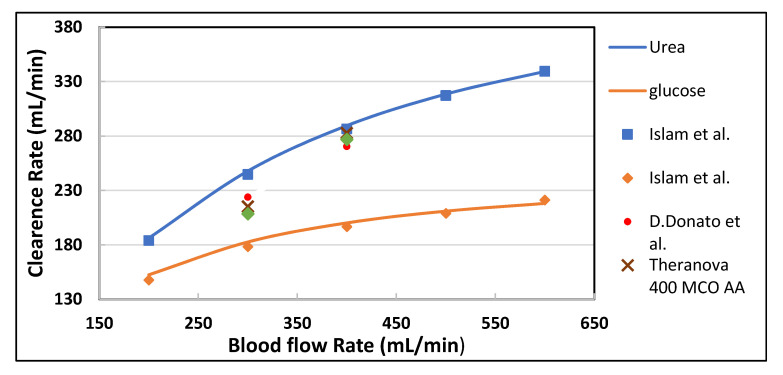
The model predicted (solid lines) vs. in vivo and in silico (symbols) solute clearances plotted against varying blood flow rate at Q_D_ = 500 mL/min.

**Figure 6:**
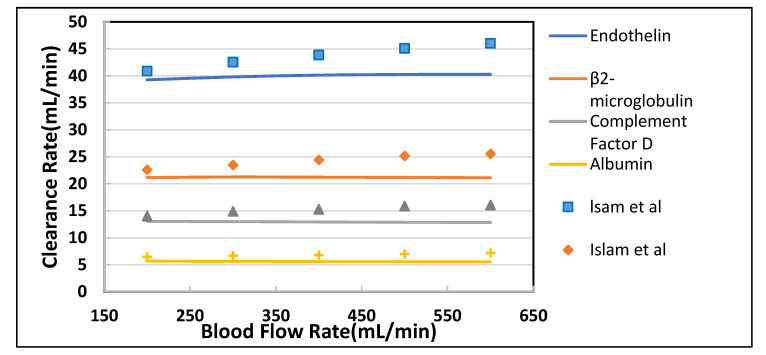
The model predicted (solid lines) vs. in vivo and silico (symbols) solute clearances plotted against varying blood flow rate at Q_D_ = 500 mL/min.

For model parameters, manufacturer data of Polyflux 210H (Gambro Dialysatoren GmbH, Germany, a subsidiary of Baxter International Inc.) was used and predicted clearance rate of different solutes were compared with Islam et al. [22], Theranova 400 MCO AA (Gambro Dialysatoren GmbH, Germany, a subsidiary of Baxter International Inc.), Polyflux 210H [23] and FX CorDiax 80 (Fresenius Medical Care, Bad Homburg, Germany) [24]. The blood flow rate was varied from 300 to 500 mL/min keeping dialysate flow rate constant (Q_D_ = 500 mL/min). In-silico and in-vivo clearance rates plotted against increasing blood flow rate, were found in good agreement. It is evident from Figure 5 and Figure 6 that the increase in blood flow rate increases the clearance of low molecular weight (LMW) solutes (urea, glucose) but does not affect the clearance of solutes with high molecular weight. The clearance rate of albumin is nearly constant. The increase in clearance with the blood flow rate can be attributed to the rise of concentration difference across the membrane. The concentration gradient across the membrane drives the transport of solutes. By increasing the blood flow rate, the concentration gradient was increased that ultimately enhance the clearance rate of solutes. On the other hand, the clearance of large size molecules shown in Figure 6 was not affected much due to the higher value of steric hindrance H and friction coefficient F(p). Due to the high value of steric hindrance H, the lesser volume is available for the large size molecules to pass through the cylindrical pore. The effect of steric hindrance H and friction coefficient F(p) was pronounced in Figure 6 while moving from β2 microglobulin to albumin due to an increase in the size of molecules.

**Figure 7:**
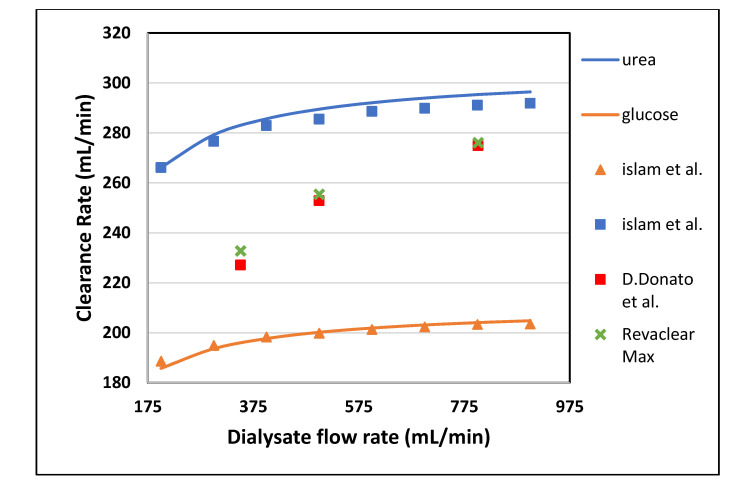
The model predicted (solid lines) vs. in vivo and in silico (symbols) solute clearances plotted against varying dialysate flow rate at Q_B_ = 400 mL/min.

**Figure 8:**
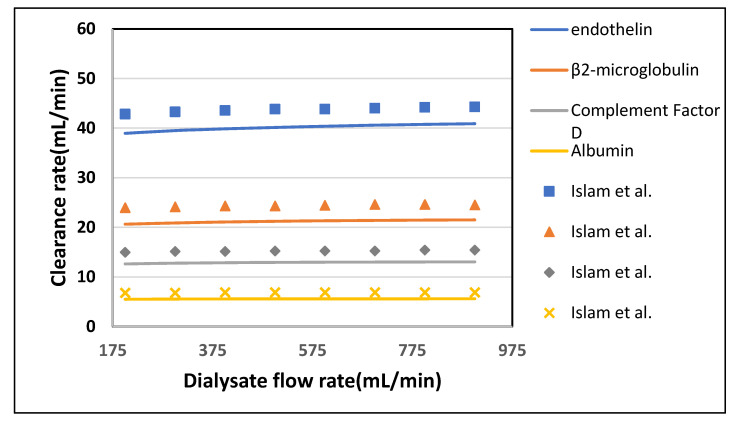
The model predicted (solid lines) vs. in vivo and silico (symbols) solute clearances plotted against varying dialysate flow rate at Q_B_ = 400 mL/min.

Table 4 shown the maximum percentage difference of this model with literature data at varying blood flow rate. Figure 7 and Figure 8 show the variation in clearance rate with dialysate flow rate. A good agreement was found between the model-predicted and Islam et al. in-silico results [22]. The trend of increase in clearance with dialysate flow is also validated by comparing with the Revaclear Max dialyzer experimental (reported in Bhimani et al. [25]) and Donato et al. in-silico results [21]. The difference between Revaclear Max and model-predicted data is due to the lack of a comprehensive dataset of module parameter values needed for model predictions. The concentration gradient also increases by increasing the dialysate flow rate Q_D_ that ultimately enhances the clearance rate of urea and glucose, as observed in Figure 7. The behavior of large size molecules in Figure 8 is similar to their behavior in Figure 6. The reason for the low diffusivity of large size molecules across the membrane despite high concentration gradient lies in the high values of steric hindrance H and friction coefficient F(p). 

For each solute, the clearance ultimately achieves a maximum, independent of the flow rate, as solutes concentration in the boundary layer C_S_^*^ approaches C_g_, gel concentration or solubility limit. This maximum clearance value is achieved faster for high molecular weight solutes. Table 5 shown the maximum percentage difference of this model with literature data at varying dialysate flow rate.

### 3.2. Effect of Module Geometry on Solute Clearances

The effect of different module dimensions on the clearance rate of solute was investigated. It was observed that fiber length, radius, and pore size have a significant impact on the clearance rate. Therefore, these parameters are discussed in detail.

#### 3.2.1. Effect of Fiber Length

**Figure 9:**
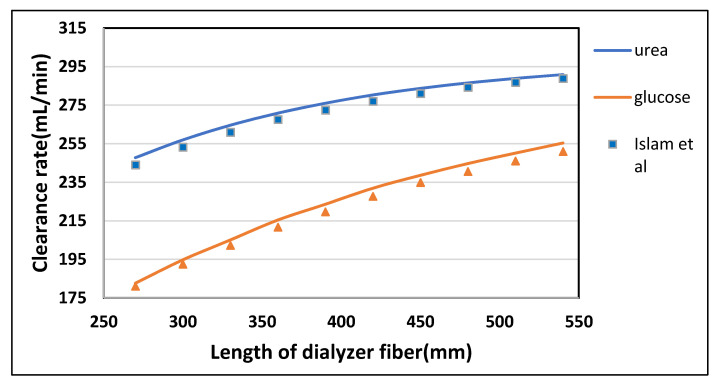
The clearance rate of low molecular weight solutes (urea, glucose) plotted against varying length of the dialyzer at Q_B_ = 300 mL/min and Q_D_ = 500 mL/min.

**Figure 10:**
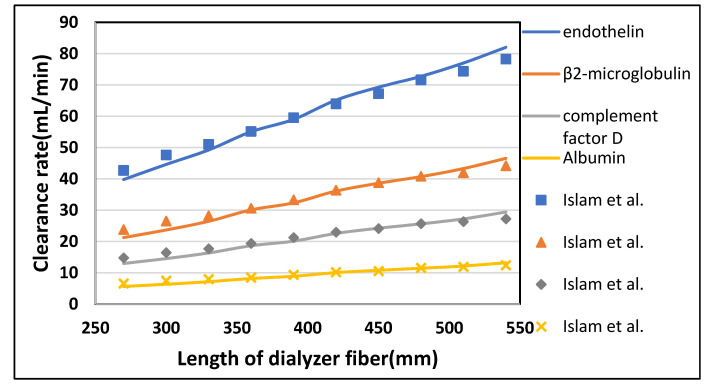
The clearance rate of high molecular weight solutes plotted against varying length of the dialyzer at Q_B_ = 300 mL/min and Q_D_ = 500 mL/min.

The fiber length was varied from 270 mm to 540 mm while keeping blood flow rate Q_B_ = 300 mL/min and dialysate flow rate Q_D_ = 540 mL/min. From Figure 9, it is evident that the clearance rate of urea and glucose rises rapidly by increasing the length of the fiber. Similarly, in Figure 10, the clearance rate of endothelin and β2-microglobulin is doubled by varying the length from 270 mm to 540 mm. This increase has to be wholly attributed to a rise in the total surface area of the hollow fibers. However, the albumin clearance is not affected much due to the large size of its molecules. Table 6 shown the maximum percentage difference of this model with literature data at varying dialyzer fiber length.

#### 3.2.2. Effect of Fiber Radius on Clearance Rate

**Figure 11:**
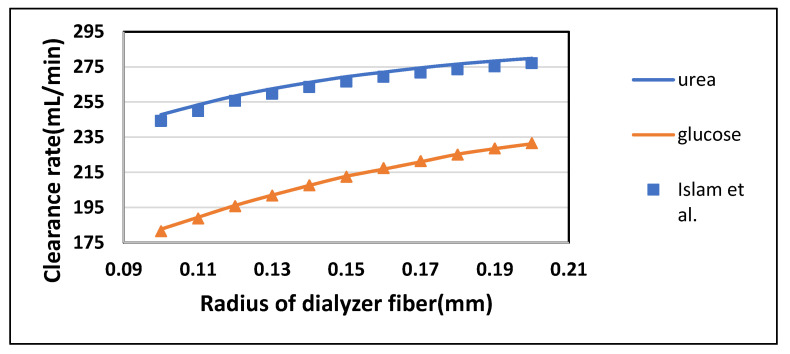
The clearance rate of low molecular weight solutes plotted against varying radius of the dialyzer at Q_B_ = 300 mL/min and Q_D_ = 500 mL/min.

**Figure 12:**
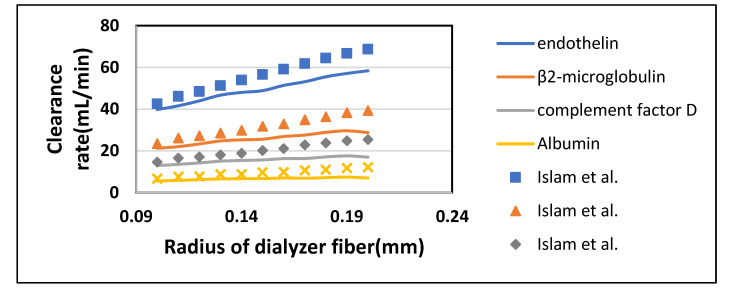
The clearance rate of high molecular weight solutes plotted against varying radius of the dialyzer at Q_B_ = 300 mL/min and Q_D_ = 500 mL/min.

Radius determines the size of the fiber cavity throughout the fiber length in an axial direction. It was varied from 0.1 mm to 0.2 mm while keeping the blood flow rate Q_B_ = 300 mL/min and dialysate flow rate Q_D_ = 500 mL/min. From Figure 11, it was observed that the clearance rate of solutes also increased by increasing the radius of fibers. This can also be attributed to an overall increase in the surface area of the membrane. Figure 12 shows that clearance of middle to large size molecules (from endothelin to albumin) also rises with an increase in the radius of the fiber. Still, the effect becomes negligible as the size of the molecule increases. Table 7 shown the maximum percentage difference of this model with literature data at a varying radius of dialyzer fiber.

#### 3.2.3. Effect of Fiber Aspect Ratio 

**Figure 13:**
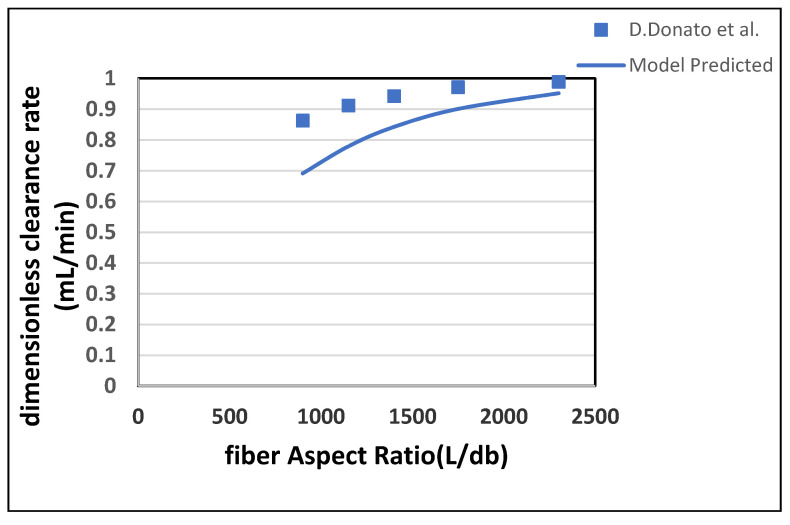
Dimensionless urea clearance rate plotted with the varying fiber aspect ratio.

The ratio between fiber length and pore diameter depicts the interplay between fiber dimensions and clearance rates. This ratio is called the fiber aspect ratio, and it is an important parameter to determine the optimum length to diameter ratio of the fiber. Figure 13 shows that as the fiber aspect ratio increases, the clearance rate also increases. Here, the fiber length is increased while keeping the radius constant. The increase in the clearance rate happens due to the rise in surface area. The difference between the values predicted by this model and Donato et al. is due to the impact of ultrafiltration, which is not included in this model. Table 8 shown the maximum percentage difference of this model with literature data at a varying aspect ratio of dialyzer fiber.

#### 3.2.4. Effect of Skin Layer Pore Size on Dimensionless Clearance

**Figure 14:**
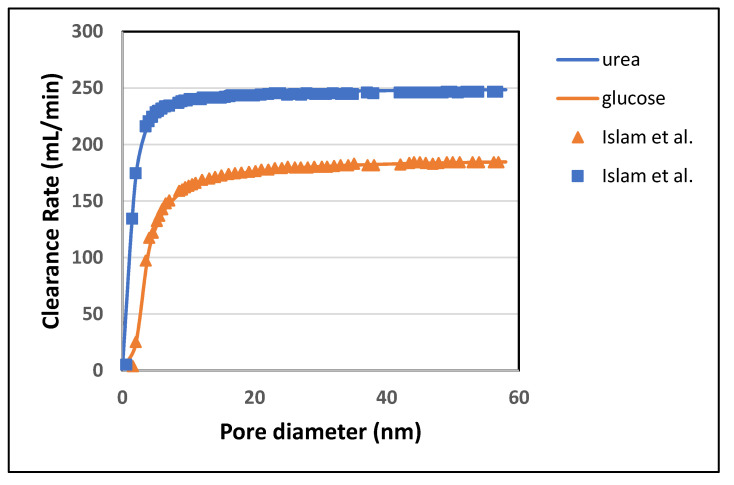
Effect of pore diameter on the clearance rate of urea and glucose.

In Figure 14, the clearance rate increases more rapidly from 10 nm to 20 nm. After 20 nm, as the pore size of the skin layer becomes equal to the middle layer, the clearance rate becomes independent of pore diameter. Since the pore size increases from the inner (skin) layer to the outer (bulk) layer, therefore inner layer, which is directly in contact with blood, plays a vital role in improving the permeability of different solutes. The skin layer has the smallest average pore size among the three layers. A small change in the skin layer pore size produces an enormous impact on the clearance of toxins. Although increasing the pore size of the inner(skin) layer enhances the clearance of small size molecules (urea and glucose), but it also adds a considerable increment to the diffusion of Albumin. This happens due to the decrease in steric hindrance S_D_ and friction coefficient F(p) of the inner layer.

**Figure 15:**
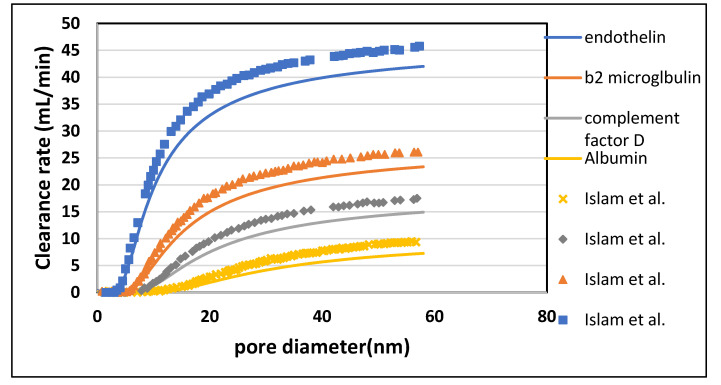
Effect of pore diameter on the clearance rate of large molecular weight (LMW) molecules.

Figure 15 shows that when pore diameter increased beyond 20 nm, the albumin molecules escaped more rapidly. However, albumin rejection is still very high in the limit of 1 to 20 nm with improved clearances of middle size molecules. This shows that increasing pore size up to 20 nm (but not beyond that) provides better clearance of the toxins, with bearable loss of albumin. Table 9 shown the maximum percentage difference of this model with literature data at a varying pore diameter of dialyzer fiber

## 4. Conclusions

Tortuous pore diffusion model (TPDM) was used to describe mass transport through the dialyzer membrane. Porosity and tortuosity were incorporated in this model to achieve a better estimation of solute clearance across the membrane. The numerical results obtained from this model were found in good agreement with the experimental results. This observation suggests that this model can be used to optimize the design and process parameters of the dialyzer module. The proposed model gave an insight into the effect of porous medium tortuosity on the diffusion of different solutes. By increasing the blood flow, the model predicted values of urea and glucose clearance were found 1.28% and 3.26% more than the Islam et al. predicted values. Similarly, the percentage increase found in urea and glucose clearance rate by increasing the dialysate flow, fiber length, and fiber radius was 1.55% and 0.4%; 1.54 and 1.86%; 1.47 and 0.6%, respectively. The clearance rate of urea was increased by 37.71% of its initial value by increasing the fiber aspect ratio. Due to the high steric hindrance H and friction coefficient F(p) the diffusion of large size molecules (i.e., endothelin, β2-microglobulin, complement factor D and albumin) do not increase much. When the pore diameter increases from 10 to 20 nm, the clearance rate of urea and glucose rise by 2.09% and 7.93% of their initial values. The results suggest that the pore diameter cannot be increased beyond 20 nm as it leads to loss of albumin molecules, which cannot be tolerated. 

## Figures and Tables

**Table 1:** Molecules that were examined in the computational analysis with their molecular weight [18] and diameter [19].

Molecule	Molecular Mass (Da)	Radius (nm)
Urea	60	0.24
Glucose	180	0.5
Endothelin	4282.8	1.30
β2-Microglobulin	11,800	1.94
Complement Factor D	24,000	2.56
Albumin	66,000	3.9

**Table 2:** A comprehensive dataset of model parameters used for model predictions [17,22].

Parameters	Values	Units
Inner radius of the fiber, r_1_	0.10	mm
Radius up to the outer layer, r_2_	0.145	mm
Radius of the concentric permeate channel, r_3_	0.210	mm
Length of the fiber, L	270	mm
Tortuosity, τ	2.27	
Inlet concentration, c_s,in_	1	mol/liter
Inlet blood flow rate, Q_B_	300	mL/min
Inlet dialysate flow rate, Q_D_	500	mL/min
Total number of fibers, N	12,000	
Porosity of skin layer, ɛ_s_	0.1	
Porosity of middle layer, ɛ_m_	0.27	
Porosity of bulk layer, ɛ_b_	0.4	
Average size of skin layer pores, d_s_	39.5	nm
Average size of middle layer pores, d_m_	450	nm
Average size of bulk layer pores, d_b_	20,400	nm

**Table 3:** Comparison of this model results with literature data [21,22].

Blood Flow Rate (mL/min)	Model Predicted	Islam et al.	Experimental Data (Polyflux 210H)	Percentage Difference of Model-Predicted and Manufacturer Data	Percentage Difference of Islam et al. and Manufacturer Data
300	247.77	244.62	281	11.82	12.94
400	289.52	286.40	339	14.59	15.51
500	318.44	317.04	378	15.75	16.12

**Table 4:** Maximum percentage difference of this model with literature data at varying blood flow rate [21].

Solutes	Blood Flow Rate (mL/min)	Model-Predicted Clearance (mL/min)	Islam et al. Clearance (mL/min)	Percentage Difference
Urea	300	247.77	244.62	1.28
Glucose	200	152.50	147.68	3.26
Endothelin	600	40.28	46.08	12.52
β2-Microglobulin	600	21.13	25.57	17.39
Complement Factor D	600	12.81	16.07	20.28
Albumin	600	5.54	7.22	23.33

**Table 5:** Maximum percentage difference of this model with literature data at varying dialysate flow rate [22].

Solutes	Dialysate Flow Rate (mL/min)	Model-Predicted Clearance (mL/min)	Islam et al. Clearance (mL/min)	Percentage Difference
Urea	900	296.45	291.84	1.57
Glucose	900	203.48	204.48	0.4
Endothelin	200	38.95	42.82	9.03
β2-Microglobulin	200	20.63	23.95	13.85
Complement Factor D	300	12.77	15.15	15.71
Albumin	400	5.56	6.89	19.28

**Table 6:** Maximum percentage difference of this model with literature data at varying dialyzer fiber length [22].

Solutes	Fiber Length (mm)	Model-Predicted Clearance (mL/min)	Islam et al. Clearance (mL/min)	Percentage Difference
Urea	270	247.77	244	1.54
Glucose	420	231.93	227.68	1.86
Endothelin	270	39.80	42.70	6.79
β2-Microglobulin	300	23.67	26.54	10.81
Complement Factor D	270	12.89	14.80	12.30
Albumin	270	5.61	6.63	15.34

**Table 7:** Maximum percentage difference of this model with literature data at a varying radius of dialyzer fiber [22].

Solutes	Fiber Radius (mm)	Model-Predicted Clearance (mL/min)	Islam et al. Clearance (mL/min)	Percentage Difference
Urea	0.1	247.77	244.18	1.46
Glucose	0.1	182.65	181.48	0.64
Endothelin	0.2	58.34	68.75	15.13
β2-Microglobulin	0.2	28.77	39.302	26.78
Complement Factor D	0.2	16.98	25.39	33.11
Albumin	0.2	6.97	12.23	42.95

**Table 8:** Maximum percentage difference of this model with literature data at a varying aspect ratio of dialyzer fiber [22].

Solutes	Fiber Aspect Ratio (-)	Model-Predicted Clearance (mL/min)	Islam et al. Clearance (mL/min)	Percentage Difference
Urea	900	0.6911	0.8625	15.52

**Table 9:** Maximum percentage difference of this model with literature data at a varying pore diameter of dialyzer fiber [22].

Solutes	Pore Dia (nm)	Model-Predicted Clearance (mL/min)	Islam et al. Clearance (mL/min)	Percentage Difference
Urea	-	-	-	-
Glucose	-	-	-	-
Endothelin	58	42.00	45.81	8.32
β2-Microglobulin	56	23.18	26.08	11.12
Complement Factor D	56	14.76	17.29	14.63
Albumin	56	7.13	9.35	23.74

## Nomenclature

$c_{s,i}$	molar concentration of the s-th solute in the i-th compartment [mol/liter]
$Cl_{s}$	Clearance rate of the j-th solute [mL/min]
$D_{e,s}$	Diffusivity of s-th solute in the i-th compartment [m^2^/s]
$d_{i}$	diameter of the i-th compartment [mm]
F	friction coefficient
$K_{s}$	overall mass transfer coefficient of s-th solute [m/s]
$k_{s,i}$	mass transfer coefficient of s-th solute in the i-th compartment [m/s]
$k_{s,mj}$	mass transfer coefficient of s-th solute in the j-th membrane layer
L	length of the fiber [mm]
N	total number of fibers [-]
N_Sh,i_	Sherwood number in the i-th compartment [-]
N_Re,i_	Reynold number in the i-th compartment [-]
p	the ratio of solute radius to the pore radius
Q_B_	the blood flow rate in the blood compartment [mL/min]
Q_D_	the dialysate flow rate in the dialysate compartment [mL/min]
$r_{1}$	the inner radius of the fiber [mm]
$-r_{2}$	the radius up to the outer layer of the fiber [mm]
$r_{3}$	the radius of the concentric permeate channel [mm]
S_D_	steric hindrance factor at pore inlet
u_i_	axial velocity in the i-th compartment [m/s]
v_i_	radial velocity in the i-th compartment [m/s]
z	axial co-ordinate [m]
**Subscript and superscript**	
B	blood
D	dialysate
e	effective
i	i-th compartment (B,m,D)
in	inlet
j	j-th membrane layer (skin, middle, bulk)
m	membrane
**Greek symbols**	
$\delta_{j}$	the thickness of the j-th membrane layer [mm]
${ɛ}_{mj}$	membrane j-th layer porosity [-]
$\mu_{i}$	fluid viscosity in the i-th compartment [kg/ms]
$\rho_{i}$	fluid density in the i-th compartment
